# Co-infection of *Phlebotomus papatasi* (Diptera: Psychodidae) gut bacteria with *Leishmania major* exacerbates the pathological responses of BALB/c mice

**DOI:** 10.3389/fcimb.2023.1115542

**Published:** 2023-01-26

**Authors:** Fariba Amni, Naseh Maleki-Ravasan, Mahmoud Nateghi-Rostami, Ramtin Hadighi, Fateh Karimian, Ahmad Reza Meamar, Alireza Badirzadeh, Parviz Parvizi

**Affiliations:** ^1^ Department of Parasitology and Mycology, School of Medicine, Iran University of Medical Sciences, Tehran, Iran; ^2^ Department of Parasitology, Pasteur Institute of Iran, Tehran, Iran

**Keywords:** leishmaniasis, *Phlebotomus papatasi*, gut bacteria, sand fly bite, pro and anti-inflammatory cytokines, pathogenesis

## Abstract

Clinical features and severity of the leishmaniasis is extremely intricate and depend on several factors, especially sand fly-derived products. Bacteria in the sand fly’s gut are a perpetual companion of *Leishmania* parasites. However, consequences of the concomitance of these bacteria and *Leishmania* parasite outside the midgut environment have not been investigated in the infection process. Herein, a needle infection model was designed to mimic transmission by sand flies, to examine differences in the onset and progression of *L. major* infection initiated by inoculation with “low” or “high” doses of *Enterobacter cloacae* and *Bacillus subtilis* bacteria. The results showed an alteration in the local expression of pro- and anti-inflammatory cytokines in mice receiving different inoculations of bacteria. Simultaneous injection of two bacteria with *Leishmania* parasites in the low-dose group caused greater thickness of ear pinna and enhanced tissue chronic inflammatory cells, as well as resulted in multifold increase in the expression of IL-4 and IL-1β and a decrease in the iNOS expression, without changing the *L. major* burden. Despite advances in scientific breakthroughs, scant survey has investigated the interaction between micro and macro levels of organization of leishmaniasis that ranges from the cellular to macro ecosystem levels, giving rise to the spread and persistence of the disease in a region. Our findings provide new insight into using the potential of the vector-derived microbiota in modulating the vertebrate immune system for the benefit of the host or recommend the use of appropriate antibiotics along with antileishmanial medicines.

## Introduction

Globally, leishmaniasis is one of the most neglected tropical and subtropical diseases, in which hosts are diseased after receiving the infectious bite of Phlebotominae sand flies infected with protozoan parasites of *Leishmania* genus (Torres-Guerrero et al., 2017). It is estimated that 0.7-1 million new cases of leishmaniasis occur annually (WHO, 2022). The disease is clinically characterized by a diverse spectrum of manifestations, varying from self-limiting cutaneous leishmaniasis (CL) to progressive and potentially fatal visceral leishmaniasis (VL) (Murray et al., 2005). Cutaneous leishmaniasis is the most common form of leishmaniasis worldwide, and virtually 95% of the cases occur in the Americas, the Mediterranean Basin, the Middle East, and Central Asia (Hepburn, 2003; WHO, 2022). As the most frequent presentation of the disease, CL causes skin lesions that evolve from nodules to painless ulcers, which can leave long-lasting scars and serious disability or stigma (Bilgic-Temel et al., 2019; WHO, 2022).

Clinical features of leishmaniasis is extremely intricate and depend on multiple factors, especially infecting parasite species (Oshaghi et al., 2009; Thakur et al., 2018; Kato et al., 2021) and their virulence factors such as lipophosphoglycan, metalloprotease GP63, and elongation factor 1 alpha (Gupta et al., 2022), as well as genetic background of the vertebrate (Sakthianandeswaren et al., 2009; Krayem and Lipoldová, 2021), immune homeostasis of the host, and vector-derived product (Serafim et al., 2021). However, balance between type 1 and type 2 immune responses, along with other regulatory mechanisms, plays an essential role in determining the outcome of leishmaniasis (Costa-da-Silva et al., 2022). Studies have signified that a set of cytokines are respectively involved in the leishmaniasis progression, and host protection entails cytokines, including TGF-β and interleukins (IL)-4, IL-5, IL-6, IL-9, IL-10, IL-27, and IL-33 and also inducible nitric oxide synthase (iNOS), interferon (IFN)-γ, tumor necrosis factor-α, IL-2, IL-7, IL-8, IL-12p40, IL-15, IL-22, and IL-23. However, the cytokines IL-1, IL-3, IL-4, IL-13, IL-17, and IL-18 have a dual role not only in the disease progression but also in host resistance (Dayakar et al., 2019). In addition, IL-4 and IL-10 have been reported to be associated with the visceralization of cutaneous *L. major* infection (Heinzel et al., 1989; Heinzel et al., 1991).

During the last few years, research has exhibited that vector-derived factors of sand fly (e.g. saliva and gut microbiota) and *Leishmania* (e.g. the promastigote secretory gel and exosomes) origin are actively involved in vector transmission and facilitate parasite survival and its establishment in the host (Serafim et al., 2021). It is well known that leishmaniasis transmission occurs in complex biological systems, including the human host, parasite, sand fly vector, and in some cases, one or more animal reservoirs (WHO, 2010). Efforts made so far toward the development of an efficient and a safe drug or vaccine against leishmaniasis have been unsuccessful or partially successful (Altamura et al., 2022; de Vries and Schallig, 2022; Ghafari and Parvizi, 2022). Hence, to constrain and finally eliminate leishmaniasis, comprehensive research programs focusing on the role of all components of the disease and their interaction are needed.

It has fully been accepted that microbiota, bacteria in particular, are inextricably linked to leishmaniasis, an issue neglected in the early theory of infectious disease causation. Interaction of microbiota with the components of a disease cycle, starting from the vector gut and continuing to the vertebrate host, can be unilateral, bilateral, or multilateral and can have adverse, neutral or beneficial effects for the partners (Maleki-Ravasan et al., 2015; Gimblet et al., 2017; Dey et al., 2018; Karimian et al., 2018; Borbon et al., 2019; Karimian et al., 2019; Campolina et al., 2020).

Once *Leishmania* parasites reach the insect’s alimentary canal, they encounter a commensal bacterial community, where multiple microbiota-parasite-host interactions determine the ultimate fate of the parasite journey (Telleria et al., 2018). Microbiota play an important role in the physiology of nutrition, digestion, and maturation of the vector’s innate immune system (Dillon and Dillon, 2004; Weiss and Aksoy, 2011). It has been shown that eliminating or changing the microbiota can alter the development of the *Leishmania* parasite in the vector’s gut (Kelly et al., 2017; Louradour et al., 2017). Gut microbiota can also affect the parasite infection by activating the innate immune pathways of the vector (Diaz-Albiter et al., 2012). Likewise, *Leishmania* infection may also lead to a prompt loss of bacterial diversity throughout the course of infection (Kelly et al., 2017).

A large number of bacteria including the members of *Enterobacter cloacae* and *Bacillus subtilis* complexes are commensal bacteria in the gut of sand flies that transmit causative agents of CL and VL in the Old and New Worlds (Oliveira et al., 2000; Hurwitz et al., 2011; Akhoundi et al., 2012; Maleki-Ravasan et al., 2015; Fraihi et al., 2017; Gunathilaka et al., 2020; Karimian et al., 2022). Both bacteria have the ability to regulate insect immune responses (Eappen et al., 2013; Heerman et al., 2015; Zhang et al., 2021) and produce secondary metabolites that show activity against insects and their harboring microorganisms (Eappen et al., 2013; Caulier et al., 2019; Zhang et al., 2021). Therefore, they probably play an essential role in sand fly vector competence for *Leishmania* parasites (Louradour et al., 2017). Consequently, *E. cloacae* and *B. subtilis* can be considered as a shuttle system to deliver, express and spread foreign inserts to be used as promising candidates for the paratransgenic approach (Hurwitz et al., 2011; Dehghan et al., 2017; Dehghan et al., 2022).

Altogether, bacterial components of the sand fly microbiota can interfere with the development of *Leishmania* parasite inside the midgut of the sand fly vector (Telleria et al., 2018) or outside the midgut, in the skin of the vertebrate (Dey et al., 2018). Regarding the first part, as mentioned above, relatively suitable studies are available, but for the second part, one survey has suggested that eliminating the vector gut microbiota or blocking the vertebrate host’s IL-1β before parasite transmission abrogates neutrophils recruited to the site of sand fly bite and declines *Leishmania* dissemination (Dey et al., 2018).

Given the above arguments, it is apparent that the bacteria in the sand fly’s gut are a perpetual companion of *Leishmania* parasites, and the consequences of concomitance of these bacteria and the mentioned parasites have not been investigated in the initiation, continuation, and termination of the infection process. Hence, a laboratory model of *Leishmania* infection was developed in BALB/c mice through needle injection of parasites with or without sand fly gut bacteria to determine the outcomes of accompanying bacteria, in terms of type and number, in the *Leishmania* wound formation and local immune responses. The results of the present study disclosed that bacterial co-infection has a profound effect on the balance of pro- and anti-inflammatory cytokine expression, thus the severity of *L. major* lesions.

## Materials and methods

### Experimental animals

Healthy female BALB/c mice (n=192), aging 4-6 weeks, were obtained from the Laboratory of Animal Sciences at the Pasteur Institute of Iran (IPI), Tehran. The mice were housed under pathogen-free and controlled conditions with 12 h light/dark cycles at 22 ± 2°C. To acclimatize to the laboratory conditions, the animals were maintained in distress-free condition for seven days. The study protocol was approved by the Ethics Commission of IPI (ethical code: IR.PII.REC.1399.027), and the use/care of animals were performed in line with the European Community (EEC Directive of 1986; 86/609/EEC), as well as the U.K. Animals Act 1986 (EU Directive 2010/63/EU for animal experiments) guidelines.

### Microbial culture and preparation

The reference strain of *Leishmania major* (MRHO/IR/75/ER) and two bacterial species of *Enterobacter cloacae* and *Bacillus subtilis* were selected for inoculum preparation. At fifth day of culture, stationary phase promastigotes of *L. major* were harvested from RPMI 1640 medium supplemented with 10% fetal bovine serum (Gibco Invitrogen, Carlsbad, CA, USA) and 100 μg/ml of penicillin-streptomycin (Biowest, USA) incubated at 25 ± 1°C. To count parasites, the cultures were centrifuged at 5,000 ×g at room temperature for 10 min and then re-suspended in 0.5% formalin following three washes with PBS. The reason for choosing these two bacterial species was their isolation from the resting, feeding and breeding environments of *P. papatasi* and their significant effects on the development of *Leishmania* (Maleki-Ravasan, in press; Maleki-Ravasan et al., 2015). Both bacteria were grown at 37°C on a Brain Heart Infusion (BHI) agar medium plate overnight. The single-grown colonies were then subcultured in BHI broth at 100 rpm at 37°C overnight. Bacterial cells were adjusted to 1.5×10^8^ CFU/ml (optical density at 600 nm, ∼0.25) according to Kaplan et al., 2012 protocol. The stock solution was serially diluted up to 1:100 and 1:10,000 to obtain 1.5×10^6^ and 1.5×10^3^ CFU/ml of each bacterial cell for high and low doses groups, respectively.

### Mice infection

Briefly, the low (1.5×10^3^ CFU/mL) and high (1.5×10^6^ CFU/mL) doses of each bacterium were suspended in 10 μL of PBS either with or without *L. major* (1.5×10^6^/ml). *Leishmania major* alone and PBS were set as controls. The suspensions were injected intradermally into the right ear pinna of mice following anesthetizing with xylazine (10 mg/kg) and ketamine (80 mg/kg) (**Table 1**).

**Table 1:** Details of bacteria (low/high doses) and *Leishmania* parasite used for inoculum preparation.

Groups (no. of mice)	Inoculum(s)	Low-dose infection		High-dose infection	
		Bacteria (CFU/mL)	Parasite (cell/mL)	Bacteria (CFU/mL)	Parasite (cell/mL)
G1 (n=12)*	Lm+Bs	1.5 × 10^3^	1.5 × 10^6^	1.5 × 10^6^	1.5 × 10^6^
G2 (n=12)	Lm+Ec	1.5 × 10^3^	1.5 × 10^6^	1.5 × 10^6^	1.5 × 10^6^
G3 (n=12)	Lm+(Bs+Ec)	1.5 × 10^3^	1.5 × 10^6^	1.5 × 10^6^	1.5 × 10^6^
G4 (n=12)	Lm	^—^	1.5 × 10^6^	—	1.5 × 10^6^
G5 (n=12)	Bs+Ec	1.5 × 10^3^	—	1.5 × 10^6^	—
G6 (n=12)	Ec	1.5 × 10^3^	—	1.5 × 10^6^	—
G7 (n=12)	Bs	1.5 × 10^3^	—	1.5 × 10^6^	—
G8 (n=12)	PBS	—	—	—	—

*Injection volume in all groups were 10 μl.

### Measurement of lesion thickness

One week after inoculation, the thickness of ear pinna was weekly measured with a digital collis for three months. The data were represented as mean ± SD of 10-12 mice/group.

### Determination of parasite burden

The number of viable parasites in the spleens and lymph nodes of the *L. major*-infected mice with or without bacteria was determined by limiting dilution assay as described previously (Titus et al., 1985). In brief, three mice in each group were killed on 31^th^ and 90^th^ days post infection (dpi). The tissues were aseptically removed and washed with PBS and then homogenized together in 1 ml of Schneider’s *Drosophila* Medium (Gibco). The homogenate was diluted in eight serial 10-fold dilutions with the same medium. In this regard, dilutions were prepared from 1:1 to 1:10,000,000 in a total volume of 1.8 ml. About 100 μl of each suspension were distributed to 96-well microtiter plates, which were covered to prevent medium evaporation and external contamination. The plates were then incubated at 25 ± 1°C for 10 days. Positive (the presence of motile parasite) and negative (the absence of motile parasite) wells were specified using an inverted microscope (Zeiss, Germany). The parasite burden was determined after logarithmic calculation of the microscopic results by ELIDA software (Taswell, 1987).

### Evaluation of cytokine expression profiles

The local expression of pro- and anti-inflammatory cytokines in mice receiving different inoculums, as stated in **Table 1**, was investigated at the beginning, middle, and end of the infection period. Three mice from each group were randomly sacrificed, and the biopsies were prepared from the inoculation sites on the right ear pinna on days 1, 31, and 90 dpi. After homogenizing the tissues of the mice, their total RNA was extracted using the TRIZOL reagent (Invitrogen) following the manufacturer’s instructions. The RNA concentrations and its purity were determined by reading A260 and A280 on a Biotek PowerWave XS Microplate Reader (Thermo Fisher Scientific, Wilmington, DE, USA). The synthesis of cDNA was accomplished with 1 μg of total RNA using iScript cDNA Synthesis kit (Pars Tous, Iran), according to the manufacturer’s recommendations. Quantitative polymerase chain reactions (qPCR) were performed on cDNAs synthesized using primers introduced in the literature (Mizobuchi et al., 2018) (**Table 2**). The investigated cytokines were consisted of IL-4, IL-10, iNOS, IL-1β, IFN-γ, and IL-12p40. All assays were carried out using 1 μl of cDNA as the template, 10 μl of SYBR Select Master Mix (Thermo Fisher Scientific), and 0.5 μl of each forward/reverse primer on the StepOnePlus Real-Time PCR System (Thermo Fisher Scientific). The qPCR results were analyzed by 2^-ΔΔCt^ methods and normalized by GAPDH. The thermal cycling conditions for the PCR included an initial denaturation of 95°C for 5 min, followed by 45 cycles of 95°C for 15 s, 60°C for 1 min, and 95°C for 15 s. Melting curve was performed at the end of the reactions.

**Table 2:** List of primers used in this study.

Gene	Forward (5′➔3′)	Reverse (5′➔3′)
*IL-4*	GGCATTTTGAACGAGGTCAC	AAATATGCGAAGCACCTTGG
*IL-10*	GCTGGACAACATACTGCTAACC	CCCAAGTAACCCTTAAAGTCCTG
*iNOS*	GTTCTCAGCCCAACAATACAAGA	GTGGACGGGTCGATGTCAC
*IL-1β*	GAAAGACGGCACACCCACCCT	GCTCTGCTTGTGAGGTGCTGATGTA
*IFN-γ*	GGCCATCAGCAACAACATAAGCG	TGGGTTGTTGACCTCAAACTTGG
*IL-12p40*	ACAGCACCAGCTTCTTCATCAG	TCTTCAAAGGCTTCATCTGCAA
*GAPDH*	CGACTTCAACAGCAACTCCCACTCTTCC	TGGGTGGTCCAGGGTTTCTTACTCCTT

### Histological investigations

At 90 dpi, the inoculated ear tissues from three mice in each group were cut and embedded in paraffin. Next, 5-μm tissue sections were prepared and stained with hematoxylin and eosin and examined under a light microscope (Zeiss, 40× objective). The presence of chronic inflammatory cells, including neutrophils, lymphocyte, and histiocytes, was imaged and analyzed using a semi-quantitative histological scoring method (Klopfleisch, 2013).

### Statistical analyses

All experiments were repeated three times. Data analyses and graph plotting were performed using GraphPad Prism software (v. 6.07). To compare multiple groups, a two-way ANOVA analysis was applied, followed by a *post hoc* test adjusted by the Tukey’s method. The Student’s t-test was also used to compare the means between two groups. A value of P<0.05 was considered statistically significant.

## Results

### Lesion thickness change

**Figure 1:**
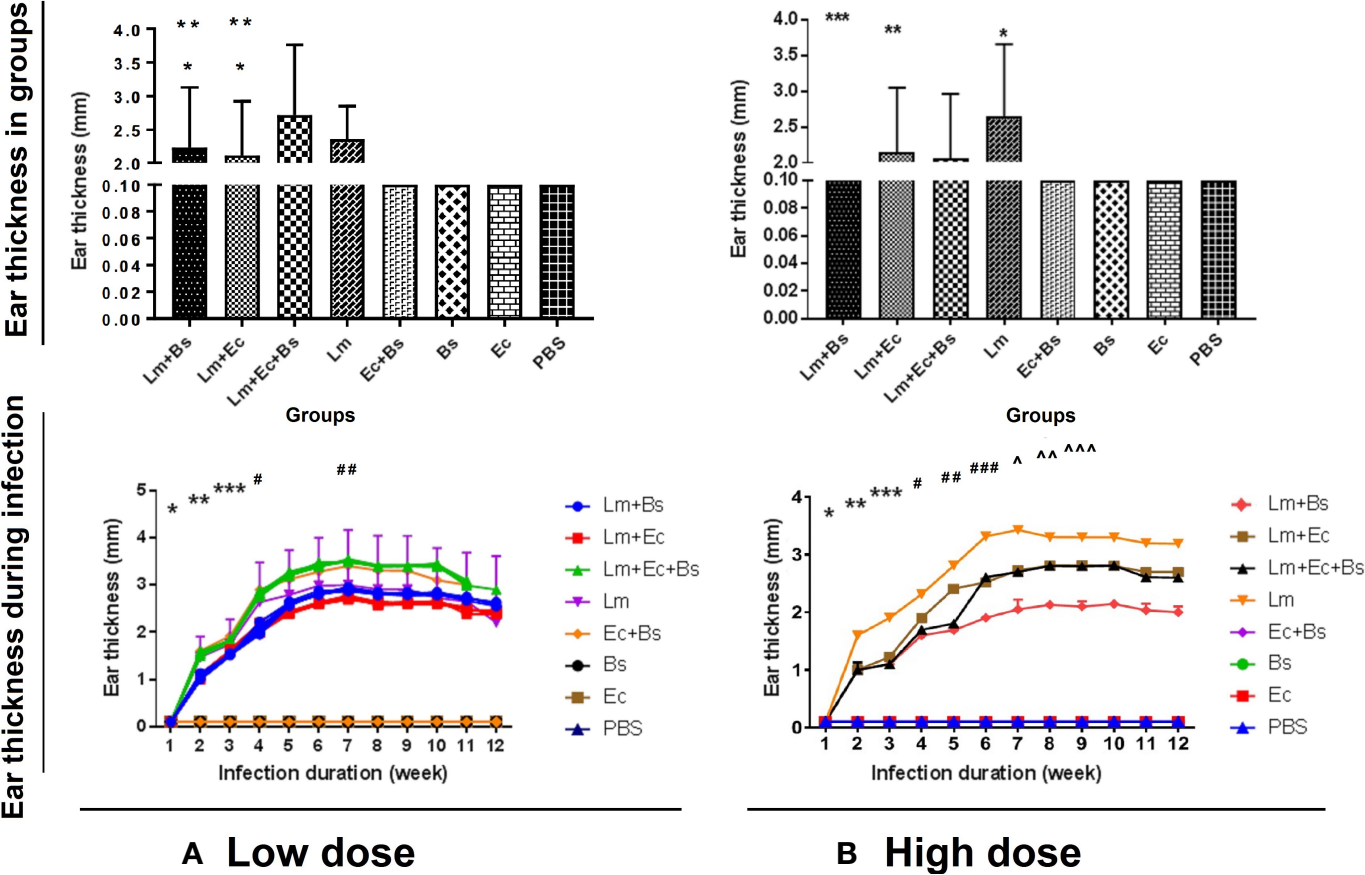
Changes in the thickness of the ear pinna of BALB/c mice in different groups (up) during infectious course (down). **(A)** Low-dose group: significance in different groups (*P<0.0001 vs. Lm; **P<0.0001 vs. Lm+(Ec+Bs) and during infectious course (*P<0.0001 vs. all; **P<0.0001 vs. all; ***P<0/0001 vs. all; #P<0.0001 vs. 1-4 and 6-10; ##P<0.0001 vs. 1-4 and 12); **(B)** High-dose group: significance in different groups (*P<0.0001 vs. Lm+(Ec+Bs), Lm+Ec, and Lm+Bs; **P<0.0001 vs. Lm+(Ec+Bs), Lm+Bs; ***P<0/0001 vs. Lm+(Ec+Bs) and during infectious course (*P<0.0001 vs. all; **P<0.0001 vs. all; ***P<0/0001 vs. all; #P<0.0001 vs. all; ##P<0.0001 vs. all; ###P<0.0001 vs. all; ^P<0.0001 vs. 1-6 and 10-12; ^^P<0.0001 vs. 1-7 and 11, 12; ^^^P<0.0001 vs. 1-6 and 11, 12).

**Figure 2:**
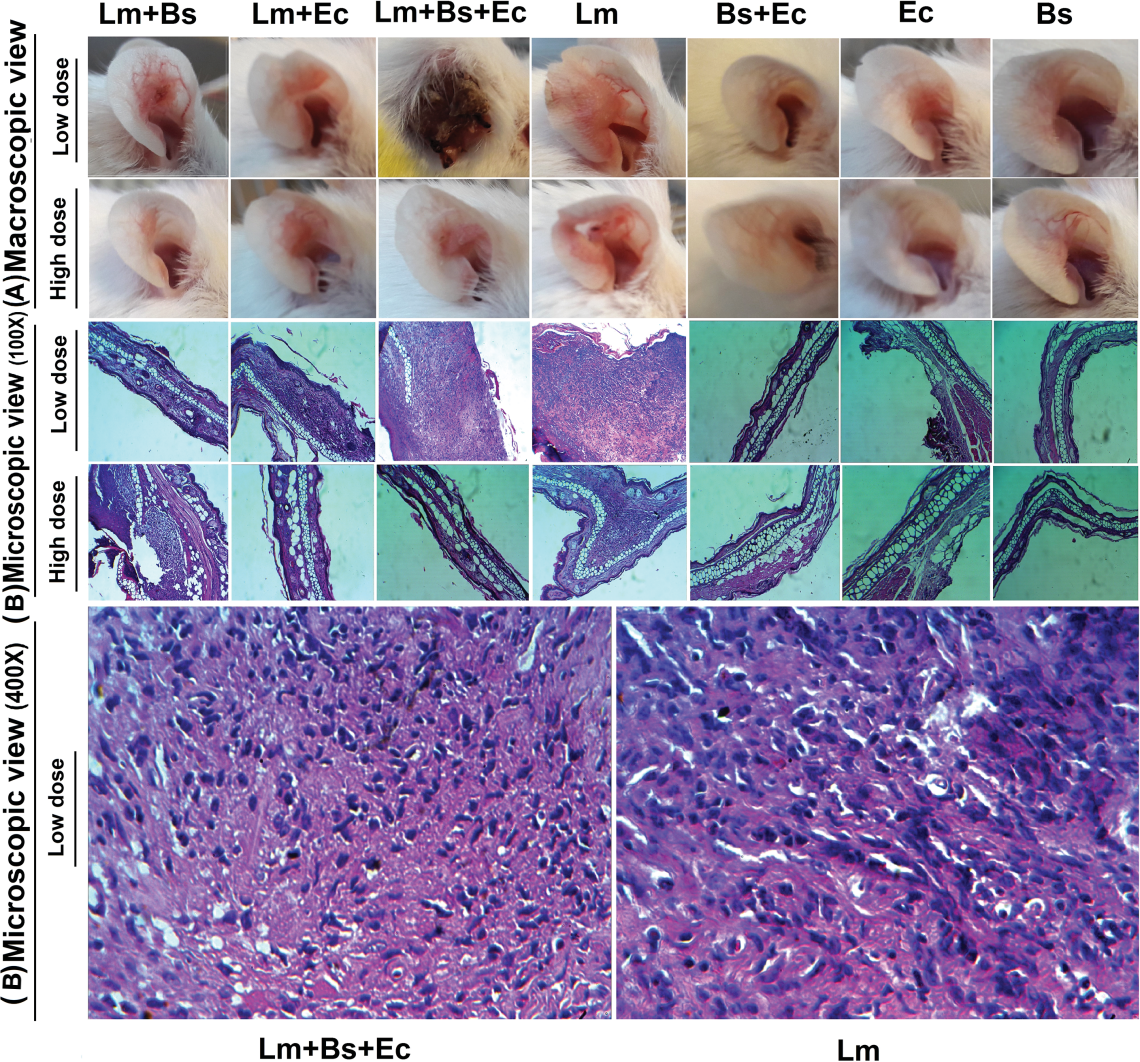
Macroscopic **(A)** and microscopic **(B)** changes in the ear pinna of BALB/c mice infected with different inoculums content at 90 dpi. The paraffin-embedded hematoxylin and eosin-stained sections of ears are illustrated by low-powered (100×) and high-powered (400×) images.

Alterations in the thickness of the ear pinna of BALB/c mice were initiated with redness and swelling one week after inoculation and peaked on the 7^th^ week so that the lesions became ulcerated (**Figures 1**, **2**). The changes were positively correlated with inoculum content. The highest thickness in low- and high-dose groups was respectively related to the groups receiving the parasite with both bacteria (**Figure 1A**; significant with the *L. major* plus *E. cloacae* or *L. major* plus *B. subtilis* bacteria, P<0.001) and the parasite alone (**Figure 1B**; significant with all groups). No changes in ear pinna thickness were observed in mice receiving only bacteria or PBS.

### Parasite burden measurement

**Figure 3:**
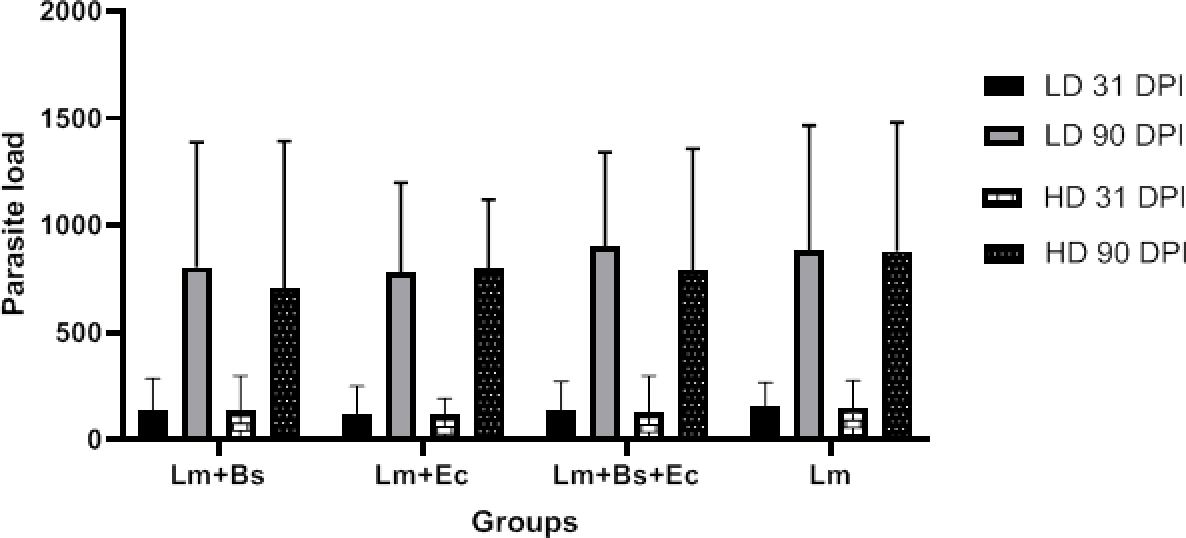
Parasite burden in the spleen and lymph nodes of BALB/c mice infected with different inoculums content at the 31/90 dpi. In all groups, P value was less than 0.001 (31 dpi vs. 90 dpi).

Measuring the parasite load of the spleen and lymph nodes at 31 and 90 dpi showed that in all (low/high doses) groups, the number of live parasites at 90 dpi was significantly higher than the 31 dpi (P<0.001); however, no significant difference was observed between the groups in both point times (**Figure 3**).

### Cytokine expression profiles

#### Low-dose group

**Figure 4:**
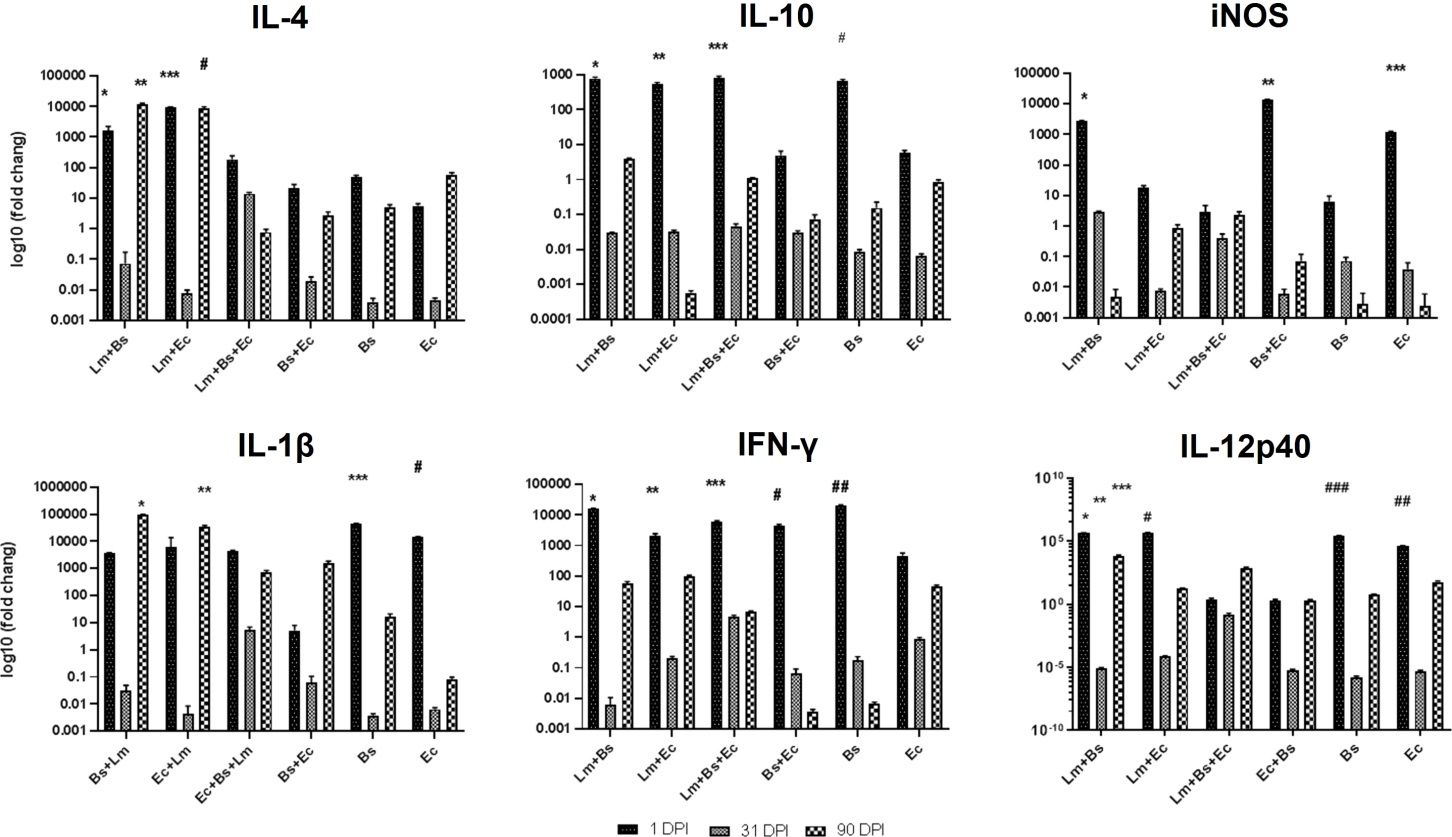
Local expression profiles of pro- and anti-inflammatory cytokines BALB/c mice infected with different inoculums in low-dose group. Symbols on the columns indicate the significant difference (P<0.0001) of the mentioned group in comparison to groups received other inoculums, as specified in the attached **Table S1**.

Pro- and anti-inflammatory cytokines generally showed increased expression at the beginning and end of the infection period compared to the middle of the period (**Figure 4**). Coinfection of bacteria and parasite caused more expression of IL-4 anti-inflammatory cytokines than the group received bacteria alone (P<0.0001). The expression of IL-10 on the first day compared to the 31^st^ and 90^th^ dpi significantly increased in all the groups (P<0.0001), except for EC/BS+EC groups. Among the mentioned cytokines, the expression level of IL-4 compared to IL-10 was significantly higher at the end of the infection period than at the beginning (P<0.0001; **Figure 4**; **Table S1**).

In case of pro-inflammatory cytokines, the simultaneous injection of two bacteria and parasites on the first day led to a significant decrease in the expression of iNOS compared to Lm+Bs, Bs+Ec, and Ec inoculums (P<0.0001). Instead, the expression of IL-1β and IFN-γ at 1 dpi in the same group was similar to other inoculums. The expression level of IL-1β was higher in the group receiving two bacteria plus parasite, as compared to the group receiving two bacteria alone at the beginning and middle of the infection period, but not significantly. In groups receiving a bacterium plus parasite, the expression level of IL-1β was significantly higher at the end of the infection period than at the middle (P<0.0001). In groups receiving two bacteria with or without parasites, the expression level of IFN-γ was significantly lower at the end of the infection period than at the beginning (P<0.0001). The expression of IL-12p40 with the injection of two bacteria alone or together with the parasite on the first dpi was significantly lower than other inoculums. The expression of iNOS and IL-12p40 showed a significant decrease at the end of the infection period compared to the beginning in Lm+Bs, Bs+Ec, and Ec groups and Lm+Bs, Lm+Ec, Bs, and Ec groups, respectively (P<0.0001; **Figure 4**).

The analysis of the mean expression of cytokines during the infection process showed that the simultaneous injection of two bacteria along with the parasite led to a significant increase in the expression of IFN-γ and IL-10, as well as a significant decrease in iNOS expression compared to the control group (P<0.0001; **Figure S1**).

#### High-dose group

**Figure 5:**
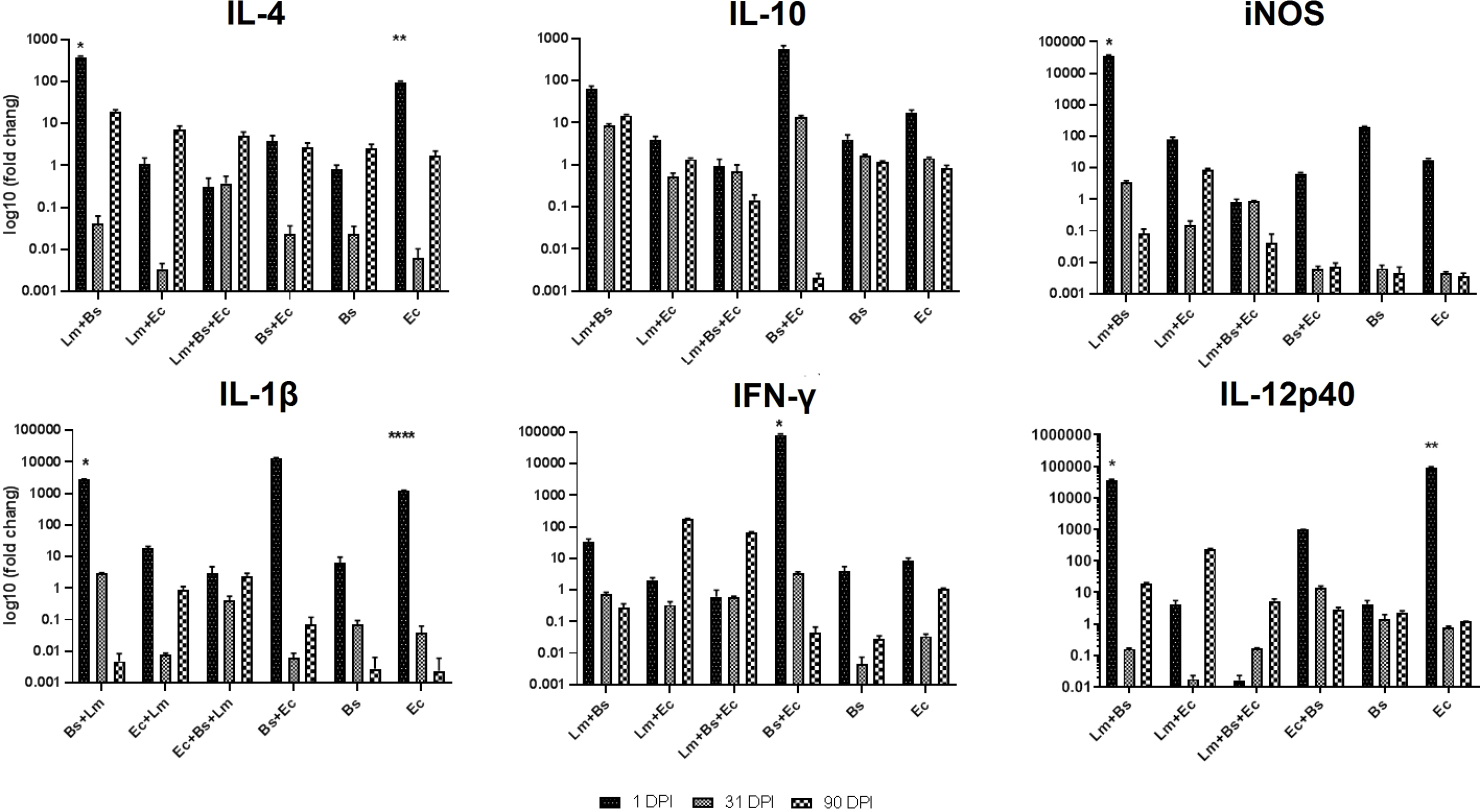
Local expression profiles of pro- and anti-inflammatory cytokines BALB/c mice infected with different inoculums in high-dose group. Symbols on the columns indicate the significant difference (P<0.0001) of the desired group with other treatments specified in the attached **Table S2**.

The expression of pro- and anti-inflammatory cytokines mostly showed fluctuations during the infection course. The anti-inflammatory cytokine IL-4 showed increased expression level at the beginning and at the end of the infection period compared to the middle; however, this variation was significant only in the Lm+Bs and Ec inoculums (P<0.0001). On the contrary, the level of Il-10 expression indicated fewer changes in all three periods. The lowest level of expression on the first day in all pro-inflammatory cytokines was found in the group receiving two bacteria with parasites. The lowest and the highest levels of IFN-γ expressions at the beginning of the infection period were observed in the groups receiving two bacteria plus parasites and in the group receiving two bacteria alone, respectively. The expression of IL-12p40 significantly decreased with the injection of two bacteria plus parasite on the first dpi compared to Lm+Bs, and Ec inoculums (P<0.0001). At the beginning of the infection period, the highest and lowest expression levels of IL-12p40 were found in groups receiving Ec and Lm+Ec+Bs, respectively (**Figure 5**).

The analysis of the mean expression of cytokines during the infection process showed that the simultaneous injection of two bacteria together with the *Leishmania* led to a significant decrease in the expression of IFN-γ, IL-1β and IL-10 compared to the control group (P<0.0001; **Figure S2**).

### Histopathological studies

**Figure 6:**
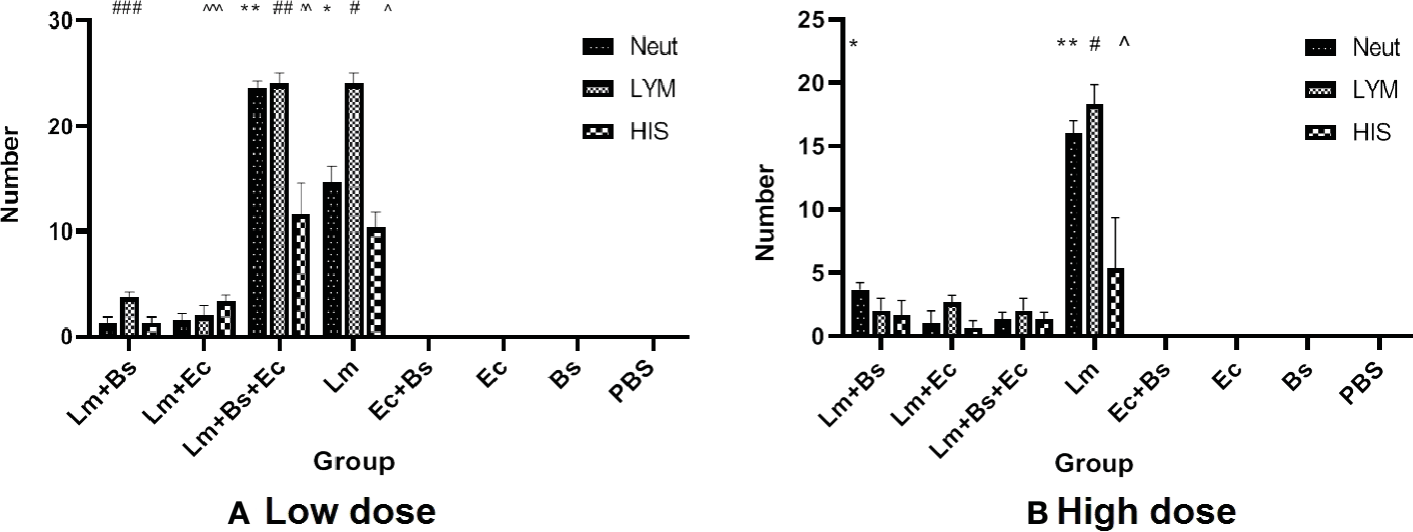
Semi-quantitative analysis of inflammatory cells of lymphocytes, histiocytes, and neutrophils in the ear pinna of mice receiving different inoculums. **(A)** Low-dose group (^*/**^ P<0.0001 vs. Bs+Ec, Bs, Ec, PBS, Lm+Ec, Lm+Bs, Lm+(Ec+Bs), and Lm; ^###/^^^^ P < 0.0001 vs. Lm+(Ec+B)s, Lm, Bs+Ec, Bs, Ec, and PBS; ^##/#^ P<0.0001 vs. Lm+Ec, Bs+Ec, Bs, Ec, and PBS; ^^^^ P < 0.0001 vs. Lm+Bs, Bs+Ec, Bs, Ec, and PBS; ^^^ P<0.0001 vs. Lm+Bs. **(B)** high-dose group (*P<0.0001 vs. Lm, Ec+BS, Ec, Bs, and PBS; ^**^ P<0.0001 vs. Lm+Ec, Lm+(Bs+Ec), Ec+Bs, Ec, Bs, and PBS; ^#/^^ P<0/0001 vs. Lm+Bs, Lm+Ec, Lm+(Bs+Ec), Ec+Bs, Ec, Bs, and PBS.

Coinfection of *L. major* with *E. cloacae* and *B. subtilis* increased the number of tissue chronic inflammatory cells at 90 dpi. The highest numbers of neutrophils in low- and high-dose groups were found in mice receiving *L. major* plus two bacteria and receiving the parasite alone, respectively. Semi-quantitative histologic scores in low-dose group showed that lymphocytes and histiocytes were predominated in both the group infected with *L. major* alone and the group infected with *L. major* plus *E. cloacae* and *B. subtilis* (**Figure 6**). Increasing the dose of bacteria did not lead to an elevation in the number of inflammatory cells in mice receiving the parasite accompanied by the bacteria.

## Discussion

The findings of the present study demonstrated that *E. cloacae* and *B. subtilis* bacteria significantly influence the infection process caused by *L. major* parasite, and the results can be used in the interpretation of the natural infection transmission through sand fly bites. Bacterial co-infection had a profound impact on the expression balance of pro- and anti-inflammatory cytokines at the beginning, middle, and end of infection course. These effects varied based on the type and dose of bacteria inoculum. From the microscopic and macroscopic point of view, the worst type of wound was observed in the group receiving low-dose *E. cloacae* and *B. subtilis* bacteria plus *L. major* parasite, leading to a decrease in the expression of iNOS and an increase in the expression of other cytokines together with raising inflammatory cells. However, the same treatment with increasing dose of bacteria showed different results in terms of wound morphology and the expression of cytokines and inflammatory cells.

There is sufficient evidence suggesting that studies of the evolutionary course of leishmaniasis, as an ancient disease, are consistent with the development of infectious disease modeling theories. At the beginning of the 20^th^ century, early studies had focused on the identification of *Leishmania* parasites as an etiological agent and also sand flies as the transmission vectors of leishmaniasis (Steverding, 2017). Later, factors other than the main partners of the disease were identified, which described the other mechanisms involved in the transmission of the disease in more detail (Serafim et al., 2021). Recently, a new multiscale model of infectious disease systems, namely the replication-transmission relativity theory, has been developed. This theory denotes that at each level of organization of an infectious disease system, pathogens must succeed at both the microscale (where pathogen replication often occurs) and the macroscale (where pathogen transmission often occurs) if they are going to spread and persist at a special level of organization of an infectious disease system (Garira, 2019). However, due to the neglect of the dynamics of the disease and the interaction between micro and macro scales, leishmaniasis still remains a major health problem in many countries, including Iran. Hence, the present study was designed and carried out in line with the multiscale replication-transmission relativity theory to show that the microbiota of the sand fly gut may affect the survival, reproduction, pathogenesis, and spread of the *Leishmania* parasite.

Lately, it has been suggested that the relationship between *Leishmania* and the microbiota may extend beyond the vector midgut. During sand fly feeding, bacteria from the insect’s gut or in the skin of the vertebrate host can enter the bite site. It has been proven that the gut microbiota of sand flies are transferred while sugar feeding (Maleki-Ravasan, in press) or are co-egested with the *Leishmania* parasite (Dey et al., 2018). Using a murine VL model of vector-transmitted *L. donovani* parasite, Dey and colleagues demonstrated that the gut microbes of the *Lutzomyia longipalpis* are entered the host skin, where they induce inflammation and IL-1β production by neutrophils. IL-1β then acts as a primary autocrine signal to attract neutrophils to the bite site. The same authors have also displayed that the microbe-induced immune response controls the downstream events governing *L. donovani* dissemination (Dey et al., 2018). Regarding the simultaneous infection of the microbiota of vertebrate skin with the *Leishmania* parasite, it has been found that this type of inoculation aggravates the disease both by promoting more inflammation and neutrophil recruitment and by increasing neutrophil apoptosis and delaying the resolution of the inflammatory response (Borbon et al., 2019).

The present research is the first study investigating the role of bacteria isolated from sand fly gut in the wound formation and local immune responses in BALB/c mice infected with *L. major*. While this study was a laboratory model, without inclusion of saliva or unculturable bacteria found in microbiota from the sand fly, its results can be used to deduce the key role of bacteria in the infectious bite of sand flies. The most important issue in our investigation was the type and the number of bacteria transmitted during the sand fly bite. Our previous study uncovered that culturable bacteria constitute a small portion of microbiota transmitted during bites (Maleki-Ravasan, in press), which should be taken into account in future studies. Therefore, tactically, the consequences of the association of a Gram-positive bacterium with a Gram-negative bacterium alongside the *Leishmania* parasite were investigated in the present study. On the one hand, the average number of parasites and bacteria egested by a sand fly is estimated to be about 1,000 and 45,000 (Maleki-Ravasan, in press; Rogers et al., 2004), respectively, and on the other hand, the number of 10^2^–10^7^ *Leishmania* parasites has been indicated to cause ulcers in an animal model (Kimblin et al., 2008; Loeuillet et al., 2016). Considering these data, the number of parasites was selected as 1.5×10^6^ and that of bacteria as 1.5×10^3^ and 1.5×10^6^ in low and high doses, respectively (**Table 1**).

The gut of insects, compared to mammals’ gut, harbors relatively fewer microbial species, but the gut of most of them contains specialized bacteria (Engel and Moran, 2013). Insects such as sand flies have a symbiotic relationship with their gut microbiota, which has become a necessary evolutionary consequence of their survival in extreme environmental conditions (Gupta and Nair, 2020). Various evolutionary processes in the insect body have led to different types of symbiotic relationships, from free living to an obligate or facultative symbiosis (Gupta and Nair, 2020). There is insufficient information on the type of symbiosis between *B. subtilis* and *E. cloacae* together and with the host insect, but evidence of their co-occurrence in the gut of eight sand fly species (Maleki-Ravasan et al., 2015; Karimian et al., 2019; Karimian et al., 2022) and their transmission while biting (Maleki-Ravasan, in press) have been provided. While these two bacteria were pre-coexisted in sand fly gut, in the present study, they first were isolated, then sub-cultured separately and finally combined while injecting into the mice, which resulted in relatively severe symptoms of *L. major*, though it may not be the case in natural settings. All these issues require more detailed and accurate investigations.

In the current study, the thickness of the ear pinna lesion in BALB/c mice was found to be bacteria dose-dependent. Thus, the highest thickness in low- and high-dose groups was related to the groups receiving the parasite plus two bacteria and the parasite alone, respectively (**Figure 1**). The results of ear lesion thickness and the abundance of tissue chronic inflammatory cells (**Figure 6**) are consistent with the findings of a recent study (Dey et al., 2018). However, the contradictory performance of high doses of bacteria in wound formation is probably due to the interaction of the Gram-positive with Gram-negative bacteria, which requires more in-depth studies.

Measurement of the parasite load during the experimental infection period showed that the number of live parasites was much higher at the end than in the middle of the infection period. In addition, there was no significant difference between the parasite loads of the groups with or without bacteria, indicating that microbiota more likely have an impact only on the pathogenesis of the parasite not on its number (**Figure 3**). The results of both ear lesion thickness and parasite load examined herein were in agreement with the Borbon et al.’s findings (Borbon et al., 2019).

In the immunology of leishmaniasis, balance between Th1 and Th 2 along with regulatory mechanisms determines the outcome of leishmaniasis. In general, Th1-type response mediates host resistance, and Th2-type response associates with disease progression in experimental infection with *L. major* in a mouse model (Dayakar et al., 2019). The results of our study showed that the local expression of pro- and anti-inflammatory cytokines in mice receiving different inoculations of bacteria changed and caused the disease to worsen. By comparing the low-dose group with high-dose group, we found that the simultaneous injection of two bacteria together with parasites causes a 35- and 970-time increase in the expression of IL-4 and IL-1β in the low-dose group. In both groups, the lowest iNOS expression was observed in the group receiving two bacteria plus parasites at 1 dpi. A significant increased expression of IFN-γ was also observed on the first day of all treatments in the low-dose group, while in the high-dose group, the elevated expression of the cytokine was detected only in the treatments receiving Bs+EC (P<0.0001). Coinfection of two bacteria with the parasite led to 260-fold increase in the expression of IL-12p40 in the low-dose group compared to the high-dose group. Moreover, the simultaneous infection of the same bacteria without parasite resulted in 328-fold increase in the expression of IL-12p40 in the high-dose group compared to the low-dose group (**Figures 4**, **5**). Also, by comparing the mean expression of cytokines in mice receiving two bacteria along with parasites compared to the control group, it was found that the expression of IL-10 is completely dose-dependent (**Figures S1**, **S2**). Therefore, as the results imply, the bacteria of the sand fly’s gut act as mice immunomodulators in adjusting the outcome of leishmaniasis.

As stated above, leishmaniasis has a complex epidemiology, and apart from main partners of the disease, various factors are responsible for the severity of the parasite’s pathogenicity. Thus, it is necessary to deeply analyze the role of neglected factors, such as microbiota, in modulating *Leishmania* pathogenesis, in order to achieve a comprehensive view of the complicated interaction of *Leishmania* parasite with its hosts. Current leishmaniasis prevention and control measures and access to valid diagnostic methods and effective treatments are insufficient. Therefore, these deficiencies could have significant implications for the disease, including increase in the incidence of leishmaniasis in the endemic foci and its neighboring localities, the spread of *Leishmania* species into new areas going unnoticed, increase in treatment failure, and the development of resistance to treatments (Berriatua et al., 2021).

## Conclusions

The results of this study suggest that the co-infection of sand fly gut bacteria with *L. major* aggravates the pathological responses of BALB/c mice. This finding gives new insight into using the capability of the vector-derived microbiota in modulating the vertebrate immune system for the benefit of the host or using appropriate antibiotics together with antileishmanial drugs. The design of the present study and the proposed model included features that were easily controlled, but there are many factors in patients - including host-specific variability - that cannot be managed and should await future studies. The data represented in the present study can be a small step to initiate a new series of studies, though it faces some limitations, such as failing to consider the systemic immune responses of mice and the role of sand fly saliva in the pathogenesis of *Leishmania* parasite. This pioneering study can be expanded to other levels of organization of leishmaniasis *via* applying advanced OMICS technologies, with the contribution of all partners of leishmaniasis. Perhaps, it is better to describe the process of wound formation in leishmaniasis from the time of the sand fly bite to the formation of nodules and wounds and even its recovery considering the role of microbiota in more detail.

## Data availability statement

The original contributions presented in the study are included in the article/**Supplementary Material**, further inquiries can be directed to the corresponding author/s.

## Ethics statement

The animal study was reviewed and approved by Ethics Commission of IPI and IUMS (ethical codes: IR.PII.REC.1399.027 and IR.IUMS.FMD.REC.1400.385).

## Author contributions

NM-R conceived and coordinated the study. NM-R, MN-R and FA designed and performed the majority of the experiments. FA, MN-R, and NM-R conducted animal studies. FA, MN-R, AB, and AM contributed to microbial culture and *Leishmania maintenance*. FK performed statistical analyses. FA, MN-R, RH, and PP contributed to real-time PCR assays and parasite burden. RH, and FA contributed to the histopathological investigations. NM-R, PP, and RH analyzed all the data. NM-R and FA wrote the paper with input from all of the authors. All authors contributed to the article and approved the submitted version.
