# Corrigendum: Co-infection of *Phlebotomus papatasi* (Diptera: Psychodidae) gut bacteria with *Leishmania major* exacerbates the pathological responses of BALB/c mice

**DOI:** 10.3389/fcimb.2023.1185912

**Published:** 2023-03-29

**Authors:** Fariba Amni, Naseh Maleki-Ravasan, Mahmoud Nateghi-Rostami, Ramtin Hadighi, Fateh Karimian, Ahmad Reza Meamar, Alireza Badirzadeh, Parviz Parvizi

**Affiliations:** ^1^ Department of Parasitology and Mycology, School of Medicine, Iran University of Medical Sciences, Tehran, Iran; ^2^ Department of Parasitology, Pasteur Institute of Iran, Tehran, Iran

**Keywords:** leishmaniasis, *Phlebotomus papatasi*, gut bacteria, sand fly bite, pro and anti-inflammatory cytokines, pathogenesis

## Incorrect Affiliation

In the published article, there was an error in affiliation 1. Instead of “Department of Parasitology and Mycology, **Faculty** of Medicine, Iran University of Medical Sciences, Tehran, Iran”, it should be “Department of Parasitology and Mycology, **School** of Medicine, Iran University of Medical Sciences, Tehran, Iran”.

The authors apologize for this error and state that this does not change the scientific conclusions of the article in any way. The original article has been updated.

