# Supplementary material for: Co-infection of Phlebotomus papatasi (Diptera: Psychodidae) gut bacteria with Leishmania major exacerbates the pathological responses of BALB/c mice
Source: Front Cell Infect Microbiol. 2023 Jan 26;13:1115542. doi: 10.3389/fcimb.2023.1115542 (PMC9909354; doi:10.3389/fcimb.2023.1115542)
Supplement: Supplementary file 1 [file DataSheet_1.docx]

Supplementary Material

**Co-infection of *Phlebotomus papatasi* (Diptera: Psychodidae) gut bacteria with *Leishmania major* exacerbates the pathological responses of BALB/c mice**

**Fariba Amni^1^, Naseh Maleki-Ravasan^2,*^, Mahmoud Nateghi Rostami^2,*^, Ramtin Hadighi^1,*^, Fateh Karimian^2^, Ahmad Reza Memar^1^, Alireza Badirzadeh^1^, Parviz Parvizi^2,*^**

**Supplementary Table 1**; Details of treatments with significant differences (P<0.0001) in the expression of pro- and anti-inflammatory cytokines with groups marked by diverse symbols in Figure 4.

| Cytokines/ Symbols | * | ** | *** | # | ## | ### |
| --- | --- | --- | --- | --- | --- | --- |
| IL4 | Lm+Bs:31,90 DPI; Lm+Ec:1,31,90 DPI,  Lm+Bs+Ec:1,31,90 DPI, Bs+Ec:1,31,90 DPI, Bs:1,31,90 DPI,  Ec:1,31,90 DPI | Lm+Bs:31 DPI, Lm+Ec:1,31,90 DPI, Lm+Bs+Ec:1,31,90 DPI, Bs+Ec:1,31,90 DPI, Bs:1,31,90 DPI, Ec:1,31,90 DPI | . Lm+Bs:31 DPI, Lm+Ec:31 DPI, Lm+Bs+Ec:1,31,90 DPI, Bs+Ec:1,31,90 DPI, Bs:1,31,90 DPI, Ec:1,31,90 DPI | Lm+Bs:31 DPI,  Lm+Ec:31 DPI, Lm+Bs+Ec:1,31,90 DPI,  Bs+Ec:1,31,90 DPI, Bs:1,31,90 DPI,  Ec:1,31,90 DPI |  |  |
| IL10 | Lm+Bs:31,90 DPI, Lm+Ec:1,31,90 DPI, Lm+Bs+Ec:31,90 DPI, Bs+Ec:1,31,90 DPI, Bs:1,31,90 DPI,  Ec:1,31,90 DPI | Lm+Bs:31,90 DPI, Lm+Ec:31,90 DPI, Lm+Bs+Ec:1,31,90 DPI, Bs+Ec:1,31,90 DPI, Bs:1,31,90 DPI, Ec:1,31,90 DPI | Lm+Bs:31,90 DPI, Lm+Ec:31,90 DPI, Lm+Bs+Ec:31,90 DPI, Bs+Ec:1,31,90 DPI, Bs:1,31,90 DPI,  Ec:1,31,90 DPI | Lm+Bs:31,90 DPI,  Lm+Ec:31,90 DPI,  Lm+Bs+Ec:31,90 DPI, Bs+Ec:1,31,90 DPI,  Bs:31,90 DPI,  Ec:1,31,90 DPI |  |  |
| INOS | Lm+Bs:31,90 DPI, Lm+Ec:1,31,90 DPI, Lm+Bs+Ec:1,31,90 DPI, Bs+Ec:1,31,90 DPI,  Bs:1,31,90 DPI,  Ec:1,31,90 DPI | Lm+Bs:31,90 DPI, Lm+Ec:1,31,90 DPI, Lm+Bs+Ec:1,41,90 DPI, Bs+Ec:1,90 DPI, , Bs:1,31,90 DPI, Ec:1,31,90 DPI | Lm+Bs:31,90 DPI, Lm+Ec:1,31,90 DPI, Lm+Bs+Ec:1,31,90 DPI, Bs+Ec:31,90 DPI,  Bs: 31,90 DPI,  Ec: 31,90 DPI |  |  |  |
| IL1b | Bs+Lm:1,31 DPI, Ec+Lm:1,31,90 DPI,Ec+Bs+Lm:1,31,90 DPI, Bs+Ec:1,31,90 DPI, Bs:1,31,90 DPI,  Ec:1,31,90 DPI | Bs+Lm:1,31 DPI, Ec+Bs+Lm:1,31,90 DPI, Bs+Ec:1,31,90 DPI, Bs:1,31,90 DPI, Ec:1,31,90 DPI | Bs+Lm:1,31 DPI, Ec+Lm:1,31 DPI, Ec+Bs+Lm:1,31,90 DPI, Bs+Ec:1,31,90 DPI, Bs:31,90 DPI,  Ec:1,31,90 DPI | Bs+Lm:1,31 DPI, Ec+Lm:1,31 DPI, Ec+Bs+Lm:1,31,90 DPI, Bs+Ec:1,31,90 DPI,  Bs:31,90 DPI, Ec:31,90 DPI |  |  |
| IFN | Bs+Lm:1,31 DPI, Ec+Lm:1,31,90 DPI,Ec+Bs+Lm:1,31,90 DPI, Bs+Ec:1,31,90 DPI, Bs:1,31,90 DPI,  Ec:1,31,90 DPI | Lm+Bs:31,90 DPI, Lm+Ec:31,90 DPI, Lm+Bs+Ec:1,31,90 DPI, Bs+Ec:1,31,90 DPI, Bs:1,31,90 DPI, Ec:1,31,90 DPI | Lm+Bs:31,90 DPI, Lm+Ec:31,90 DPI, Lm+Bs+Ec:31,90 DPI, Bs+Ec:1,31,90 DPI, Bs:1,31,90 DPI, Ec:1,31,90 DPI | Lm+Bs:31,90 DPI, Lm+Ec:31,90 DPI, Lm+Bs+Ec:31,90 DPI, Bs+Ec:31,90 DPI,  Bs:1,31,90 DPI,  Ec:1,31,90 DPI | Lm+Bs:31,90 DPI, Lm+Ec:31,90 DPI, Lm+Bs+Ec:31,90 DPI, Bs+Ec:31,90 DPI, Bs:31,90 DPI, Ec:1,31,90 DPI |  |
| IL12p40 | Lm+Bs:31,90 DPI, Lm+Ec:1,31,90 DPI, Lm+Bs+Ec:1,31,90 DPI, Bs+Ec:1,31,90 DPI, Bs:1,31,90 DPI,  Ec:1, 31,90 DPI | Ec+Lm:1 DPI,  Bs:1 DPI, Ec:1 DPI | Ec+Lm:1 DPI,  Bs:1 DPI | Ec+Lm:31,90 DPI, Bs+Ec+Lm:1,31,90 DPI, Ec+Bs:1,31,90 DPI, Bs:1,31,90 DPI,  Ec:1,31,90 DPI | Ec+Lm:31,90 DPI, Bs+Ec+Lm:1,31 DPI, Ec+Bs:1,31,90 DPI, Bs:31,90 DPI,  Bs:31 DPI . Ec:90 DPI | Ec+Lm:90 DPI, Bs+Ec+Lm:1 DPI, Bs+Ec+Lm:31 DPI, Ec+Bs:1 DPI,  Ec+Bs:31 DPI, Bs+Ec+Lm:90 DPI, Ec+Bs:90 DPI, Ec+Lm:31 DPI,  Bs:31 DPI,  Bs:90 DPI,  Ec:31 DPI,  Ec:90 DPI |

**Abbreviations:** Bs: *Basilus subtilis*; dpi: day post infection; Ec; *Enterobacter cloacae*; IFN-γ: interferon gamma; IL: interleukin; iNOS: inducible nitric oxide synthase; Lm: *Leishmania major*

**Supplementary Table 2**; Details of treatments with significant differences (P<0.0001) in the expression of pro- and anti-inflammatory cytokines with groups marked by diverse symbols in Figure 5.

| Cytokines/ Symbols | * | ** | *** | # |
| --- | --- | --- | --- | --- |
| IL-4 | Lm+Bs:31,90 DPI, Lm+Ec:1,31,90 DPI, Lm+Bs+Ec:1,31,90 DPI,  Bs+Ec:1,31,90 DPI, Bs:1,31,90 DPI,  Ec:1,31,90 DPI | Lm+Bs:31 DPI, Lm+Ec:1,31,90 DPI, Lm+Bs+Ec:1,31,90 DPI,  Bs+Ec:1,31,90 DPI, Bs:1,31,90 DPI,  Ec:1,31,90 DPI | Lm+Bs:31 DPI,  Lm+Ec:31 DPI, Lm+Bs+Ec:1,31,90 DPI,  Bs+Ec:1,31,90 DPI, Bs:1,31,90 DPI,  Ec:1,31,90 DPI | Lm+Bs:31 DPI,  Lm+Ec:31 DPI, Lm+Bs+Ec:1,31,90 DPI, , Bs+Ec:1,31,90 DPI,  Bs:1,31,90 DPI,  Ec:1,31,90 DPI |
| IL-10 | Lm+Bs:1,31,90 DPI, Lm+Ec:1,31,90 DPI, Lm+Bs+Ec:1,31,90 DPI,  Bs+Ec:31,90 DPI, Bs:1,31,90 DPI,  Ec:1,31,90 DPI |  |  |  |
| iNOS | Lm+Bs:31,90 DPI, Lm+Ec:1,31,90 DPI, Lm+Bs+Ec:1,31,90 DPI,  Bs+Ec:1,31,90 DPI, Bs:1,31,90 DPI, Ec:1,31,90 DPI |  |  |  |
| IL-1b | Bs+Lm:31,90 DPI, Ec+Lm:1,31,90 DPI, Ec+Bs+Lm:1,31,90 DPI,  Bs+Ec:1,31,90 DPI, Bs:1,31,90 DPI,  Ec:1,31,90 DPI | Bs+Lm:31,90 DPI, Ec+Lm:1,31,90 DPI, Ec+Bs+Lm:1,31,90 DPI,  Bs+Ec:31,90 DPI, Bs:1,31,90 DPI,  Ec:1,31,90 DPI | Bs+Lm:31,90 DPI, Ec+Lm:1,31,90 DPI, Ec+Bs+Lm:1,31,90 DPI, |  |
| IFN | Lm+Bs:1,31,90 DPI, Lm+Ec:1,31,90 DPI, Lm+Bs+Ec:1,31,90 DPI,  Bs+Ec:31,90 DPI, Bs:1,31,90 DPI,  Ec:1,31,90 DPI |  |  |  |
| IL-12p40 | Bs+Lm:31,90 DPI, Ec+Lm:1,31,90 DPI, Bs+Ec+Lm:1,31,90 DPI,  Ec+Bs:1,31,90 DPI, Bs:1,31,90 DPI,  Ec:1,31,90 DPI | Bs+Lm:31,90 DPI, Ec+Lm:1,31,90 DPI, Bs+Ec+Lm:1,31,90 DPI,  Ec+Bs:1,31,90 DPI,  Bs:1 DPI,  Bs:31,90 DPI,  Ec:31,90 DPI |  |  |

**Abbreviations:** Bs: *Basilus subtilis*; dpi: day post infection; Ec; *Enterobacter cloacae*; IFN-γ: interferon gamma; IL: interleukin; iNOS: inducible nitric oxide synthase; Lm: *Leishmania major*


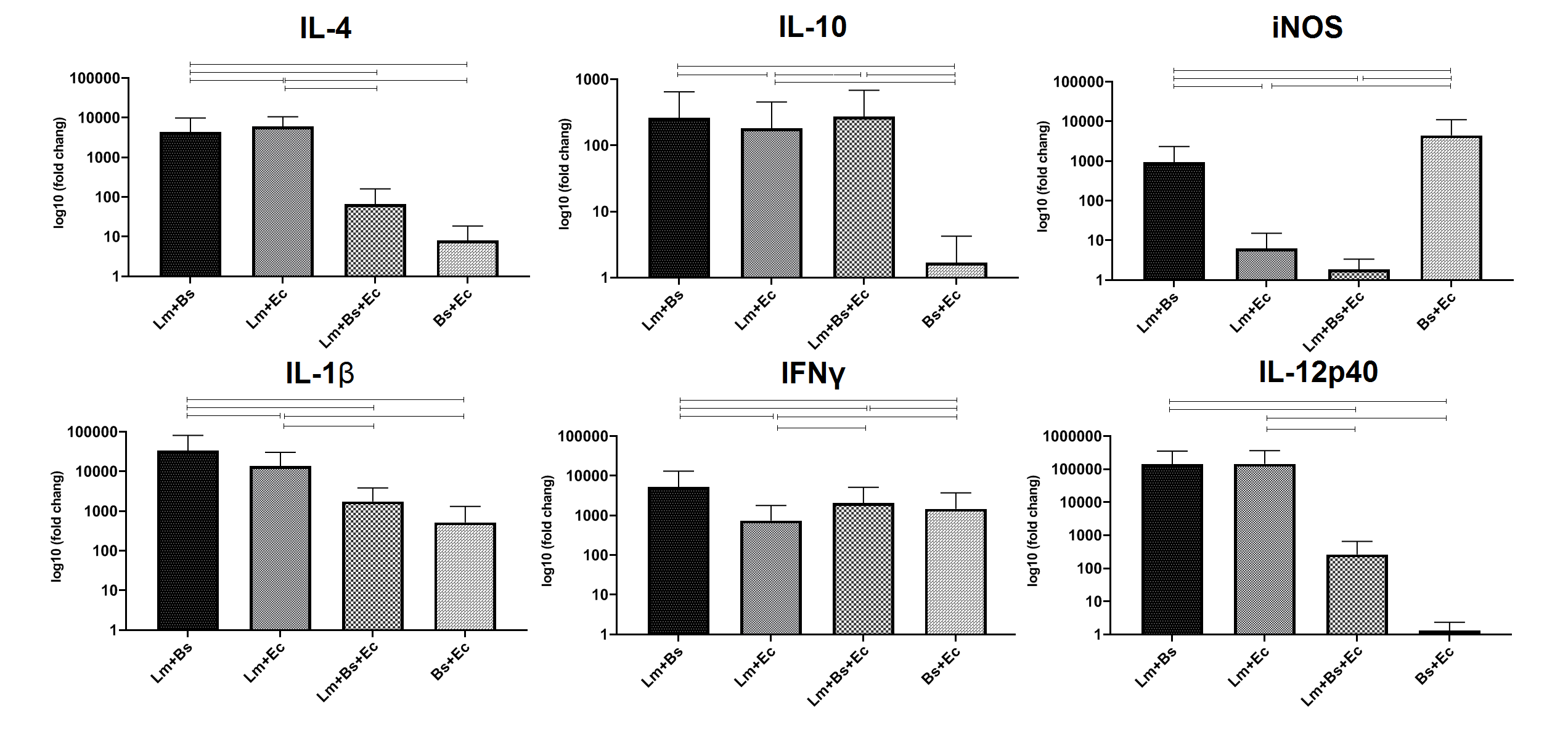


**Supplementary Figure 1**; The mean expression of pro- and anti-inflammatory cytokines in BALB/c mice receiving different inoculums in low-dose group. Lines on the columns indicate the significant difference (P<0.0001) between the groups under comparison.

**
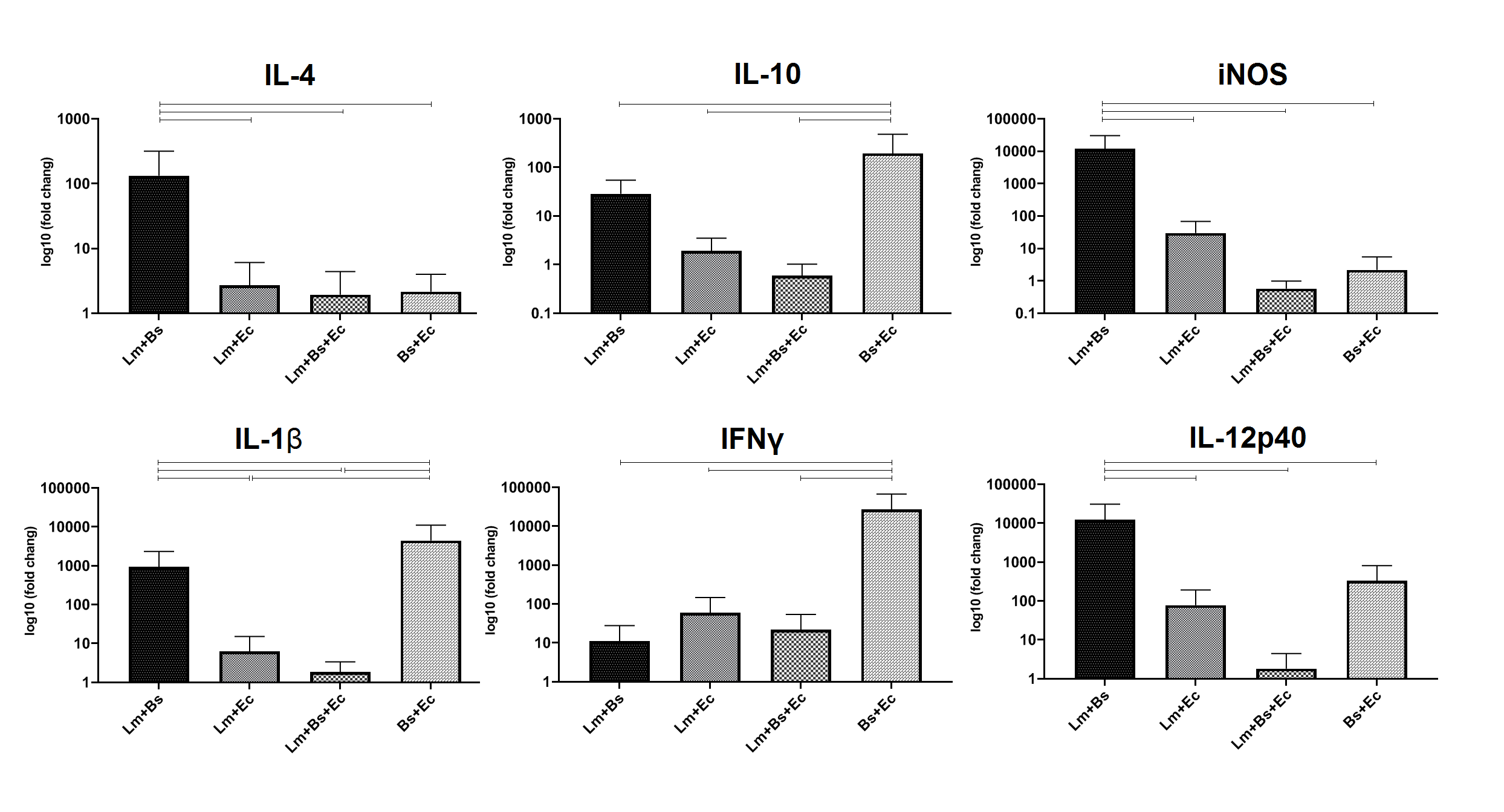
**

**Supplementary Figure 2;** The mean expression of pro- and anti-inflammatory cytokines in BALB/c mice receiving different inoculums in high-dose group. Lines on the columns indicate the significant difference (P<0.0001) between the groups under comparison.
